# Accuracy Report on a Handheld 3D Ultrasound Scanner Prototype Based on a Standard Ultrasound Machine and a Spatial Pose Reading Sensor

**DOI:** 10.3390/s22093358

**Published:** 2022-04-27

**Authors:** Radu Chifor, Tiberiu Marita, Tudor Arsenescu, Andrei Santoma, Alexandru Florin Badea, Horatiu Alexandru Colosi, Mindra-Eugenia Badea, Ioana Chifor

**Affiliations:** 1 Department of Preventive Dentistry, University of Medicine and Pharmacy Iuliu Hatieganu, 400083 Cluj-Napoca, Romania; chifor.radu@umfcluj.ro (R.C.); mebadea@umfcluj.ro (M.-E.B.); ioana.chifor@umfcluj.ro (I.C.); 2 Computer Science Department, Technical University of Cluj-Napoca, 400114 Cluj-Napoca, Romania; santoma.va.andrei@student.utcluj.ro; 3 Chifor Research SRL, 400068 Cluj-Napoca, Romania; tudor.arsenescu@chiforvision.com; 4 Anatomy Department, University of Medicine and Pharmacy, 400006 Cluj-Napoca, Romania; alexandru.badea@umfcluj.ro; 5 Department of Medical Education, Division of Medical Informatics and Biostatistics, Iuliu Hatieganu University of Medicine and Pharmacy, 400349 Cluj-Napoca, Romania; hcolosi@umfcluj.ro

**Keywords:** 3D ultrasonography, freehand 3D ultrasound scanner prototype, quantitative 3D reconstruction evaluation, 2D image segmentation, pose sensor, coordinate measuring machine

## Abstract

The aim of this study was to develop and evaluate a 3D ultrasound scanning method. The main requirements were the freehand architecture of the scanner and high accuracy of the reconstructions. A quantitative evaluation of a freehand 3D ultrasound scanner prototype was performed, comparing the ultrasonographic reconstructions with the CAD (computer-aided design) model of the scanned object, to determine the accuracy of the result. For six consecutive scans, the 3D ultrasonographic reconstructions were scaled and aligned with the model. The mean distance between the 3D objects ranged between 0.019 and 0.05 mm and the standard deviation between 0.287 mm and 0.565 mm. Despite some inherent limitations of our study, the quantitative evaluation of the 3D ultrasonographic reconstructions showed comparable results to other studies performed on smaller areas of the scanned objects, demonstrating the future potential of the developed prototype.

## 1. Introduction

Ultraportable imaging equipment, such as handheld sonographic machines with wireless systems, shows adequate accuracy, performance and good quality of images compared to high-end sonographic machines [1]. The low cost and the handling of such portable sonographic machines might raise an increased interest among clinicians, especially in emergency medicine departments, but having diagnostic imaging competence may be decisive in driving the correct therapeutic decision. Ultrasound quality is operator dependent and subjective to interpretive error; in order to successfully integrate this technology into their clinical practices, physicians must be familiar with the normal and abnormal appearance of tissues [2]. Conventional two-dimensional (2D) ultrasound imaging is a powerful diagnostic tool in the hands of an experienced user; however, 2D ultrasound remains clinically underutilized and inherently incomplete, with the output being very operator dependent. Providing a simple and inexpensive method of acquiring complete volumetric 3D ultrasound images, with sensed pose information and intuitive feedback displayed to the user, is an important step towards solving the problem of operator dependence. The usefulness of the real-time 3D US was demonstrated by a large variety of clinical applications, further indicating its role and significance in the fields of medical imaging and diagnosis [3]. Its cost is relatively low in comparison to CT and MRI, no intensive training or radiation protection are required for its operation, and its hardware is movable and can potentially be portable [4]. Previous studies showed that a volume measurement using the 3D US devices has a similar accuracy level to that of CT and MR [5]. 

The accuracy of ultrasound medical systems seems to depend significantly on settings, as well as on phantom features, probes and investigated parameters. The relative uncertainty due to the influence of probe manipulation on spatial resolution can be very high (i.e., from 10 to more than 30%), and field of view settings must also be taken into account [6]. However, previous studies have shown that an ultrasound scanner was able to scan teeth with an accuracy similar to that of conventional optical scanners when no gingiva was present [7], and ultrasonography is suitable for periodontal imaging [8], even if it requires an extremely high accuracy, due to the size and complexity of the investigated anatomical elements.

Three-dimensional ultrasounds may store volumes describing the whole lesion or organ. A detailed evaluation of the stored data is possible by looking for the features that were not fully appreciated at the time of data collection, or by applying new algorithms for volume rendering, in order to glean important information [9]. Three-dimensional imaging could be an advantage, especially in the education of future surgical generations. Recent studies have shown that the modern 3D technique is superior to 2D, in an experimental setting [10]. The manual guidance of the probe makes reproducible image acquisition almost impossible. Volumetric data offer the distinct advantage of covering entire anatomical structures, and their motion paths can then be used for automated robotic control [11]. 

The aim of this study was to develop and evaluate a highly accurate 3D ultrasound scanning method. The main requirements imposed on the new scanning method were the free hand architecture of the scanner and the high accuracy of the reconstructions, ranging between the computer tomography reconstructions and optical scans, as well as no movement restrictions during scanning. 

The main contributions of this paper are as follows: it documents a quantitative evaluation of a freehand 3D ultrasound scanner prototype, comparing the ultrasonographic reconstructions with the CAD (computer-aided design) model of the scanned object to determine the accuracy of the result; proposes a semi-automatic segmentation method of the raw US images (region growing-based segmentation, followed by morphological filtering and a customized upper contour (envelope) extraction); proposes an evaluation method by comparing the 3D ultrasound reconstructed object with the original 3D CAD model, by computing the mean distance and standard deviation after their alignment.

## 2. Materials and Methods

**Figure 1:**
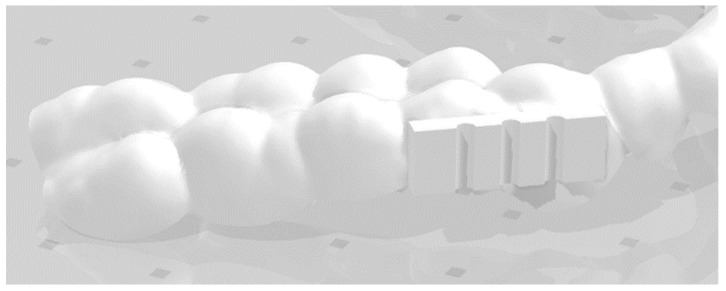
The scanned CAD/CAM manufactured mouth guard, the attachment exhibiting both curved and rectilinear contours and positive and negative relief with regular and irregular multiple concavities and convexities.

A 3D ultrasound scanner prototype based on a 2D standard ultrasound machine and a spatial pose reading sensor was developed using Vinno 6 (Suzhou, China) equipment with a high frequency (10-23 MHz) and a small aperture (12.8 mm) linear transducer (X10-23L) and as a pose reading sensor, an articulated measurement arm (Evo 7, RPS Metrology (Sona/Italy). The articulated measurement arm RPS EVO 7 accuracy was 34 μm. According to the technical specifications, the following information was obtained: “It has no need for calibrations or warm-up time, thanks to its extremely reliable mechanical and electronical design, the automatic temperature compensation and the lightweight structure.” The transducer was attached to the articulated arm (coordinate measuring machine, CMM). The spatial and temporal calibration of the employed devices were performed using proprietary algorithms. After calibration, a CAD/CAM manufactured object, used as a phantom, was immersed in a water tank and scanned 6 consecutive times. The CAD/CAM manufactured object was a custom mouth guard, simulating a dental arch, having attached an object with regular contours and planar surfaces, exhibiting both right angles and concave surfaces (Figure 1). The mouth guard was manufactured using DATRON D5 Linear Scales (Darmstadt, Germany,), with an accuracy of ±5 μm according to the technical specifications and PMMA as the material.

Every scanning procedure generated between 452 and 580 bi-dimensional consecutive ultrasound images (Table 1, second column), with a mean scanning time of approximately 14.3 s (the frame rate was 33 frames/second). The ultrasound scanning plane cross-sectioned the object transversally. The scanning procedure started each time at the last molar, going in mesial direction, a 6 teeth area and then backwards, in distal direction, back to the starting point. A total number of 2840 bi-dimensional ultrasound images were acquired and used for the accuracy evaluation of the 3D ultrasound reconstructions. 

### 2.1. Measuring and Verifying the CAD/CAM Manufactured Object, the Mouth Guard, Using a Method with Known and Determined Measurement Error (Intraoral Optical Scanning Method)

The mouth guard was scanned using a TRIOS 3, 3Shape (Denmark) intraoral scanner with an accuracy (trueness) of 6.9 ± 0.9 μm, according to the technical specifications. Using the protocol described below in chapter 2.5 and the CloudCompare open-source software (CCOSS) for evaluating the freehand 3D ultrasound scanner prototype, the mean distance and standard deviation were calculated for the optical scan of the mouth guard aligned with the original STL project, after adjusting the scale of the two 3D objects. 

### 2.2. Semiautomatic Segmentation of the 2D Ultrasound Images

**Figure 2:**
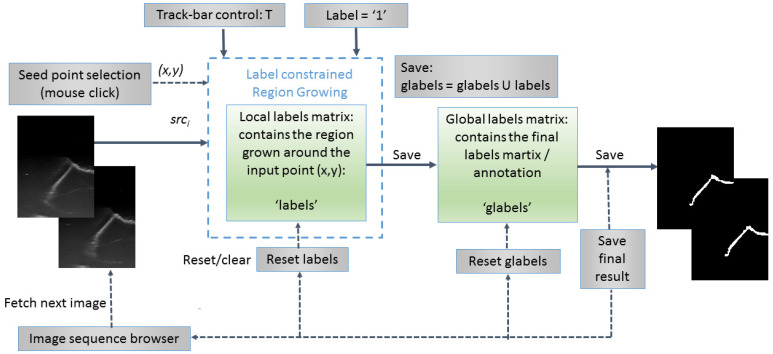
Flowchart of the semiautomatic label constrained region growing (RG)-based segmentation algorithm.

An original semiautomatic segmentation tool for the 2D US images was developed using a customized region growing-based segmentation algorithm (Figure 2). In the process, the user was supposed to click on seed points that were “grown” by iteratively adding neighboring pixels with similar intensities. The algorithm was customized in such a way that the already labeled pixels were not considered. The similarity predicate was controlled by a threshold (T), tunable by the user using a track-bar control and the result (the local grown region) was visible on the fly for any instant position of the track-bar. In general, the initial value for T should be chosen between 2σ and 3σ, where σ is the standard deviation of the Gaussian distribution of the regions of interest (ROIs) computed on some sample image patches and is application dependent. For the current application, the initial value of T was set to T ≈ 15, since σ was estimated to σ ≈ 5 for a set of samples of whitish ROIs, corresponding to the mouth guard surface regions. There was also available the option of applying morphological-based post-processings (dilation followed by erosion), in order to fill in the holes occurring after the segmentation process. Once the user (which should be a qualified/specialized operator and in this case a dentist specialized in dental ultrasonography) was satisfied with the result (criteria were as follows: maximization of the smoothness and continuity of the upper envelope of the mouth guard in each 2D image/section), the local grown region (local labels matrix) was appended to the global grown region (global labels matrix), which stored the final segmentation result in the form of a binary image.

The region growing algorithm is based on the breadth-first search (traversal) algorithm of graphs [12] and uses a queue structure (FIFO list) for optimal implementation. The grown process of each region was constrained to the selected label; therefore, the implementation can be used out-of-the-box for multi-label annotation of more complex anatomical structures in medical imaging. For the current purpose of segmenting the outer surface of the mouth guard, only one label was used (variable label was set to 1 in the segmentation and morphological post-processing algorithms) and a binary result image was obtained. The pseudocode of the proposed labeled constrained region growing-based segmentation algorithm is presented below (Algorithm 1).
**Algorithm 1** Label Constrained Region Growing1: 2: 3: 4: 5: 6: 7: 8: 9: 10: 11: 12: 13: 14: 15: 16: 17: 18: 19: 20: 21: 22: 23: 24: 25: 26: 27: 28: 29: 30: 31: 32: 33: 34:**procedure** Grow(*src*; *local_labels*; *label* = *1*; *x*; *y*; *T; applyMorph*)        *h* ← src height        *w* ← src width        *Q* ← [ ]        *W* ← 3        *d* ← W/2        *avgColor* ← average(src(*y* − *d*: *y* + *d*; *x* − *d*: *x* + *d*))        *d* ← 1        *N* ← 1        *Q:append*((*y,x*))        **if** *labels*(*y,x*) = 0 **then**
                *labels*(*y,x*) = *label*        **end if**        **while** *Q* is not empty **do**                *oldest* ← *Q.pop()*                **for** *m* ← −*d* to *d*, *n* ← *−d* to *d* **do**                        *i* ← *oldest.y + m*                        *j* ← *oldest.x + n*                        **if** (*i,j*) inside of *src* **then**                                *color* ← src(*i,j*)                                **if** |*color – avgColor*| < *T* and *labels(i,j)* = *0* and g*labels(i,j)* = *0* **then**                                        *labels(i,j)* ← *label*                                        *Q.append(i,j))*                                        *avgColor* ← *(avgColor × N +color)/(N + 1)*                                        *N* ← *N + 1*                                                        end if                        **end if**                **end for**        **end while**        *dst* ← *labels*        **if** *applyMorpho* = *true* **then**                *dst* ← Dilate*(dst*,*R*,*label)*                *dst* ← Erode*(dst*,*R*,*label)*        **end if**        **return**
*dst* **end procedure**> Empty queue > Averaging window size > No. of pixels in the region > If pixel is unlabeled > Take out the oldest element from the queue > Search across its neighbors and add them to the queue if they are not labeled and are similar in terms of color with the region > Update the average color of the region > Convert the local labels matrix into the destination image > Post-process the result by morphological operations

### 2.3. Morphological Post-Processing

An optional step of the segmentation algorithm was to perform morphological post-processing [13] in order to refine each resulted segment (grown region), mainly for filling up the small holes that occur in the segmented process. For this purpose, a dilation (Algorithm 2), followed by an erosion (Algorithm 3), was applied with a circular structuring element of adjustable radius. The implementation of the algorithms was adapted to the following proposed label constrained paradigm: the foreground pixels were dilated and eroded at the label level. The two complementary morphological operations were applied in pairs with the same structuring element (in terms of size and shape), in order to not alter the area and the overall shape of the segmented regions.
**Algorithm 2** Label Constrained Dilation1: 2: 3: 4: 5: 6: 7: 8: 9: 10: 11: 12: 13: 14: 15: 16: 17: 18: 19: 20: 21:**procedure** Dilate(*src*, *R*, *label* = *1)*        dst ← *copy(src)*
        *h* ← img height        *w* ← img width        **for** *i* ← *R* to *h* − *R* − *1*, *j* ← *R* to *w* − *R* − *1* **do**                **if** *src(i,j)* = *label* **then**                        **for** *m* ← *−R* to *R*, *n* ← −*R* to *R*
**do**                                **if** R > 2 **then**                                        *radius* ← $\sqrt{m^{2}+n^{2}}$                                        **if** *radius* < *R* and *src(i + m, j + n)* = 0 **then**                                                *dst(i + m,j + n)* ← *label*                                        **end if**                                **else**                                        **if** *src(i + m*; *j + n) = 0* **then**                                                *dst(i + m*; *j + n)* ← *label*                                        **end if**                                **end if**                            **end for**                    **end if**        **end for**        **return**
*dst* **end procedure**> Clone source image into the destination > Image scan with safety border > Apply dilation using a circular structuring element of radius R (R > 2) only on pixels with the specified label. All pixels in the neighborhood masked by the structuring element are marked with the current label > If R ≤ 2, the structuring element has a square shape

**Algorithm 3** Label Constrained Erosion1: 2: 3: 4: 5: 6: 7: 8: 9: 10: 11: 12: 13: 14: 15: 16: 17: 18: 19: 20: 21: 22: 22: 22:**procedure** Erode(*src*, *R*, *label* = *1)*        dst ← *copy(src)*
        *h* ← img height        *w* ← img width        **for** *i* ← *R* to *h − R − 1*, *j* ← *R* to *w − R − 1* **do**                *frontier* ← *false*                **if** *src(i,j)* = *label* **then**                        **for** *m* ← *−R* to *R*, *n* ← *−R* to *R*
**do**                                **if** R > 2 **then**                                        *radius* ← $\sqrt{m^{2}+n^{2}}$
                                        **if** *radius* < *R* and *src(i + m, j + n)* = 0 **then**                                                *frontier* ← *true*                                        **end if**                                **else**                                         **if** *src(i + m*; *j + n) = 0* **then**                                                *frontier* ← *true*                                        **end if**                                **end if**                        end for                **end if**                **if** *frontier* ← *true* **then**                        *dst(i,j)* ← *0*        **end for**        **return**
*dst* **end procedure**> Clone source image into the destination > Image scan with safety border > Apply erosion using a circular structuring element of radius R (R > 2) only on pixels with the specified label. If there is a background pixel in the neighborhood masked by the structuring element, a flag (*frontier*) is set > If R ≤ 2, the structuring element has a square shape > If a flag is set, the current pixel is removed from the result

### 2.4. Upper Envelop/Contour Extraction of the Segmented Objects

Before 3D reconstruction could be applied, the upper/outer envelope of the segmented objects from the 2D binary images had to be extracted in the form of a contour. This contour should correspond to the surface of the mouth guard observed in each 2D US image. The Algorithm 4 is presented below. First, the external contours of the segmented binary objects were detected and drawn as a binary image in the *findExternalContours* function using the following OpenCV [14] methods: *findContours* and *drawContours*. Then, the binary contours image was scanned column by column, from top to bottom and the first vertical sequence of white pixels from each column was stored in the destination image. This approach also dealt with cases of vertical contour segments in the upper envelope.

**Algorithm 4** Find Upper Envelope 1: 2: 3: 4: 5: 6: 7: 8: 9: 10: 11: 12: 13: 14: 15: 16:**procedure** FindEnvelope(*src)*        *contours* ← *findExternalContours(src)*
        *h* ← img height        *w* ← img width        *dst* ← *(h*,*w*,*0)*        **for** *j* ← 0 to *w − 1* **do**                **while** *contours(i,j)* = 0 and *i < h − 1* **do**                        *i* ← *I + 1*
                **end while**                **while** *contours(i,j)* = 255 and *i < h − 1*
**do**                        *dst(i,j)* ← *255*                        *i* ← *I + 1*                        **break**
                **end while**
        **end for**        **return**
*dst* **end procedure**> Detect external contours in the binary source image and store them (as white pixels) in the *contours* image > Create a black destination image > Scan the binary contour image *contours* on columns > Skip the first vertical sequence of black pixels > Store the first vertical sequence of white (object/contour) pixels in the destination image *dst* > At the end of the sequence, break the for loop (j ← j + 1, i ←0) 

**Figure 3:**
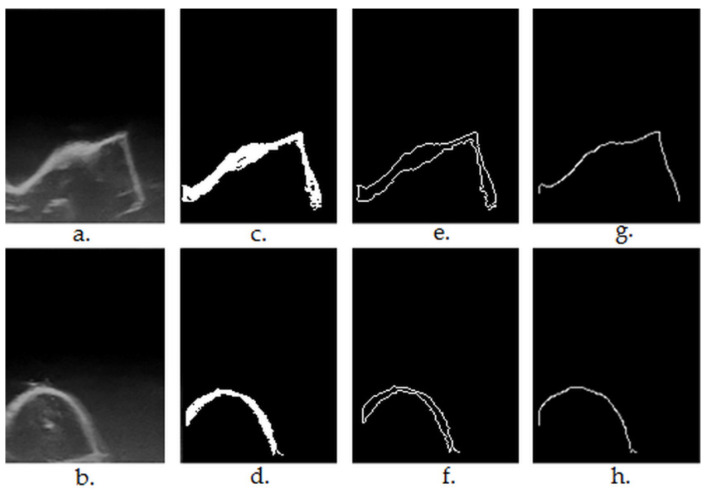
(**a**,**b**)—original ultrasound frames in greyscale ((**a**)—first premolar, regulated shaped object having plane surfaces, right angles; (**b**)—lateral incisor); (**c**,**d**)—results after region growing-based segmentation and morphological post-processing (Algorithms 1–3); (**e**,**f**)—results after contour extraction (*findExternalContours* function in Algorithm 4); (**g**,**h**)—results after upper contour (envelope) extraction (Algorithm 4).

The step-by-step results of the segmentation Algorithms 1–4 are shown in Figure 3.

### 2.5. Generating 3D Ultrasound Reconstructions 

Data acquisition and 3D reconstruction were performed using the 3D US scanner prototype and the software developed by Chifor Research’s team. After the US data were acquired, each frame was paired or matched with the sensor’s readings. The spatial coordinates and orientation of each frame were determined through the time and spatial calibration processes [15].

The 3D reconstruction was performed by introducing the scan planes corresponding to the raw 2D ultrasound images into a tridimensional space. This was carried out by using a series of rotations and translations performed in several stages, as described below. The first step was to create a 3D space to hold the voxels corresponding to the pixels in the image. The next step was to apply a calibration matrix transformation to the 2D frame corresponding to this 3D space. The third step applied the final pose transformation on the output of the previous step, in order to finalize the positioning of the original 2D frame in the corresponding 3D space allocated for it in the beginning. This final transformation represented a bijection between the points in the 2D frame and their 3D correspondents. Finally, the intensity of the original pixel in the 2D frame was assigned to the corresponding voxel in the 3D space. The previous steps were repeated for each acquired 2D ultrasound frame, until all the 2D frames were represented in the 3D space [16].

The segmented envelop/contour points from each 2D US scan were used to mask the 3D points associated with each scan and to generate the 3D point cloud corresponding to the mouth guard’s surface, which was further used for the quantitative evaluation of the reconstruction algorithm.

### 2.6. Evaluating the Accuracy of the 3D Ultrasound Reconstructions

The accuracy of the 3D virtual reconstruction, obtained by the ultrasound scanning of the CAD/CAM manufactured phantom, was evaluated by comparing it with the standard STL project, designed and used for its execution. The alignment, scaling and statistical analysis of the distances between the 3D points of the 3D ultrasound reconstruction point cloud and the reference object, the STL project, were performed using the CloudCompare open-source software (CCOSS). The CCOSS statistically analyzed the distances between the ultrasound point cloud and the STL object after the objects were spatially aligned. 

After ultrasound scanning using the developed prototype and generating the 3D reconstruction of the segmented mouth guard’s surface, the obtained point cloud was aligned with the CAD project. The mean deviation (distance) of the 3D ultrasound reconstruction from the reference model was calculated, as well as the standard deviation of the distances using CCOSS. 

The calculation of the mean distance and the standard deviation was computed during cloud point alignment to the reference point cloud or mesh, as part of the alignment algorithm. This was done in two stages. The first stage was a rough alignment, giving a rough value for the RMS (root mean square) index, representing the square root of the mean value of the squared distances *d_i_*, as described by Equation (1) [17], which is as follows:

(1) $$\mathrm{RMS}=\sqrt{\sum \left(d_{i}^{2}\right)/n}$$

where *d_i_*^2^ is the squared distance between the reconstructed 3D points and corresponding CAD model points, computed over *n* points.

Once the rough alignment was completed and its corresponding 4 × 4 transformation matrix was calculated so that the two point clouds were moved and scaled into proximity according to at least 3 corresponding points on their surface, the second step of the alignment was performed, providing a fine tuning of the RMS value, by incrementally moving and scaling the two point clouds in order to minimize the RMS. At the end of this process, the corresponding standard deviation and mean distance, which were proportionally correlated with the RMS, were calculated [18,19].

## 3. Results

The virtual alignment with the reference object (the mouth guard STL project) and the statistical accuracy evaluation were performed on six consecutive 3D ultrasound reconstructions, acquired using the handheld 3D ultrasound scanner prototype.

### 3.1. Measuring and Verifying the CAD/CAM Manufactured Object, the Mouth Guard, Using a Method with Known and Determined Measurement Error (Intraoral Optical Scanning Method)

**Figure 4:**
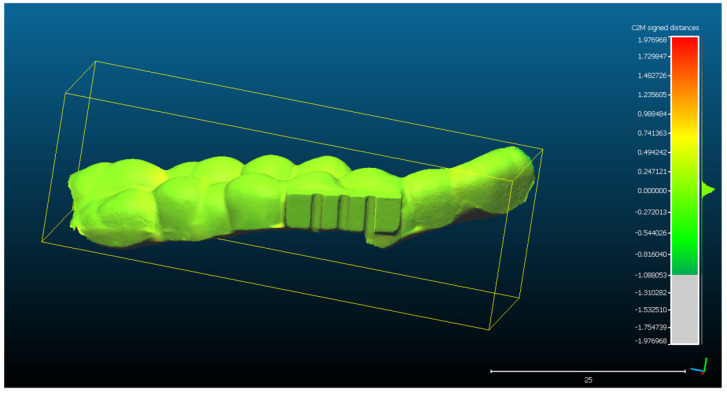
The spatial distribution of the 128,570 measurements between the optical scan of the mouth guard with the reference object (CAD project STL format) after alignment. The distribution of the alignment errors is figured in the form of a colored Gaussian shape on the right side of the color code bar. In light green are the measurements close to 0.

The alignment errors measured for the optical scan aligned with the CAD project STL of the mouth guard (Figure 4) are as follows: mean distance of 13,65 μm and standard deviation of 117.14 μm. The measured distances are represented in a Gaussian characteristic symmetric “bell curve” shape and most of the measurements are close to 0, as one can observe in the righthand section of Figure 4.

**Figure 5:**
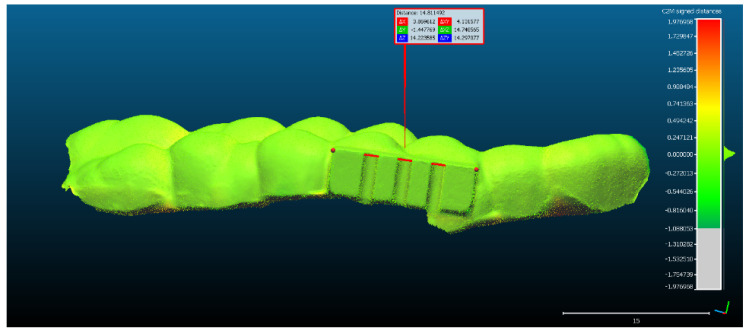
The scaling of the optical scan of the mouth guard, after it had been aligned with the reference object.

The recalculation of the mean distance and the standard deviation according to the adjusted scale (15 mm = 14.81 mm) can be observed in Figure 5, generated by CCOSS after aligning the two objects with the following errors: mean distance of 13.83 μm and standard deviation of 118.64 μm

### 3.2. Preparing the 2D Ultrasonographic Images, Performing 3D Ultrasound Reconstructions and Aligning Them with the Reference Object for Statistical Analysis

For scan 1: The original 2D ultrasound images have been segmented without extracting the contours. Subsequently, the 3D reconstruction was performed based on the semi-automatically segmented 2D images and the spatial position reading data related to each 2D frame, resulting in a point cloud of 272,189 3D points.

**Figure 6:**
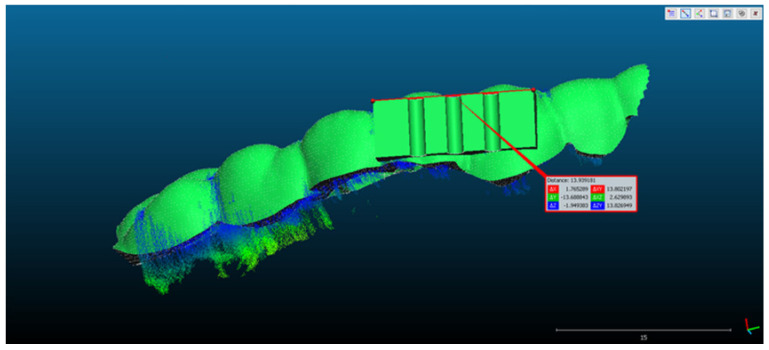
The scaling of the point cloud after it had been aligned with the reference object.

**Figure 7:**
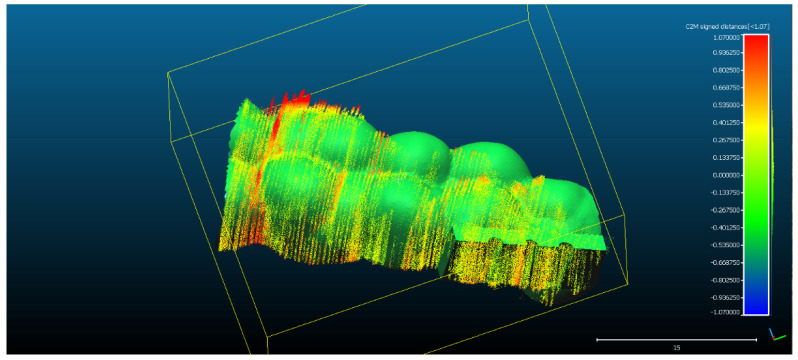
The spatial distribution of the 272,189 3D ultrasonographic points compared to the reference object (CAD project STL format) after alignment. The distribution of the alignment errors is figured in the form of a colored Gaussian shape on the righthand side of the color code bar.

**Figure 8:**
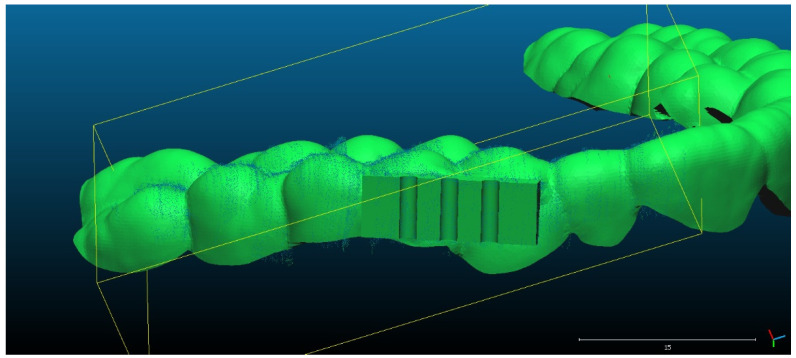
Scan 2 aligned with the reference object using CloudCompare software.

**Figure 9:**
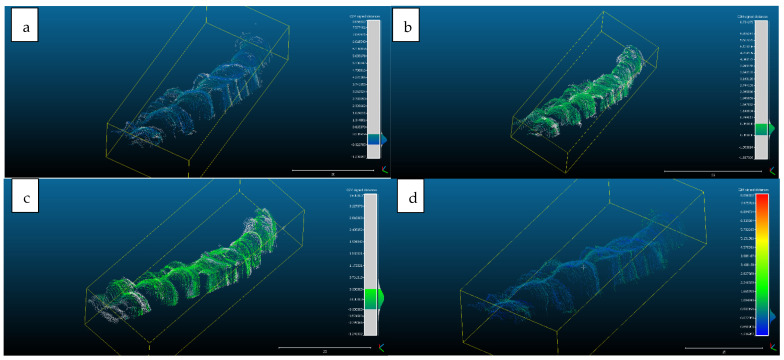
Distribution of the alignment distances/errors of the 3D ultrasonographic points from the scanned reference object after alignment in CCOSS: (**a**) Scan 2—aligned with STL reference object. The farthest points are colored in gray, alignment errors are evenly distributed along the scanned object (observe the blueish distances’/errors’ distribution on the righthand side of the figure); (**b**) Scan 3—aligned with the reference STL object (observe the greenish distances’/errors’ distribution between the 3D points and reference object on the righthand side of the figure); (**c**) Scan 4 aligned with STL reference object (3D points distances’/errors’ spatial distribution in green on the righthand side of the figure); (**d**) Scan 5—aligned with STL reference object (3D points distances’/errors’ spatial distribution in blueish on the righthand side of the figure).

- The rectangular landmark, used for scaling the objects, measures in real world 15 mm in length. Its length in CCOSS after alignment was 13.939. Thus, 1 mm length in real world equaled 1.07 in CCOSS (Figure 6).

- The spatial distribution of the 272,189 3D ultrasonographic points was compared to the reference object (CAD project in STL format) after alignment. The distance errors of the 3D ultrasonographic points were uniformly distributed, meaning that the reconstruction respected the shape of the scanned object (Figure 7). The mean distance of the 3D ultrasonographic points from the reference object was 0.033 mm and the standard deviation equaled 0.387 mm (Table 1). The deviations were most probably due to the artifacts and to the noise in the 2D ultrasound original frames.

- Scan 2. After segmentation of the 2D images, the contours were extracted, before reconstructing the 3D object. The total number of 3D ultrasonographic points (masked by the segmented contours) was significantly lower (46,537) compared to the scan 1 (279,189 3D points as they were obtained without extracting the contours); the obtained 3D point cloud was aligned with the reference object (STL project), as shown in Figure 8, and the computed mean distance was 0.031 mm and the standard deviation was 0.287 mm (Figure 9a and Table 1). The deviations/errors were isolated to certain areas, probably due to the artifacts in some of the 2D original frames.

- For Scans 3 to 5, the alignment errors are presented in Figure 9b–d. The mean distance between the 3D points of the ultrasonographic reconstructions (obtained by masking the 3D point cloud with the segmented contours) and the reference scanned object varied in the range between 0.019 mm to 0.05 mm (Table 1).

### 3.3. Quantitative Evaluation by Statistical Error Analysis

The mean and standard deviations of the distances/errors of the 3D ultrasonographic points from the scanned reference object after alignment in CCOSS are presented in Table 1.

The advantage of the method proposed in the present study is that the scanner prototype has six axes freedom of movement during scanning. The accuracy of the reconstruction was not influenced by the length of the reconstructed area. As one can observe in Figure 9a–c, there is a homogeneous distribution of the ultrasonographic 3D points situated at more than 350 microns from the reference, colored in grey, probably due to the artifacts in the 2D ultrasonographic images. If those artifacts had been due to 3D reconstruction errors, the 3D object would have been distorted, with spatially concentrated errors. 

In a normal distribution (which can be assumed based on the large number of measurements), approximately 95% of the deviations of the virtual model from the reference model range within the average +/− two standard deviations. In addition, approximately 99% of the deviations of the virtual model from the reference model range within average +/− three standard deviations.

## 4. Discussion

The aim of the current study has been reached, by developing and evaluating a highly accurate 3D ultrasound scanning method. 

The verification of the 3D ultrasound scanned object confirmed that the shape and the size of the scanned object is very close to the technical specification range of the CAD/CAM process, and also confirmed that the method used to appreciate the accuracy of the 3D ultrasound prototype is reliable. Datron D5, used for the manufacturing the mouth guard, has an accuracy of 5 μm and Trios 3 from 3Shape, used to optical scan the mouth guard, has an accuracy of 6.9 μm and 0.9 μm, resulting in a 12.8 μm possible error according to the technical specification of the two devices, because the errors can cumulate.

A previously published low-cost volumetric ultrasound imaging method has been developed by Herickhoff et al. [20], using the augmentation of bidimensional systems generating freehand 3D ultrasounds via probe position tracking. The method allowed a variety of scanning patterns (e.g., linear translation normal to the image plane or panoramic sweep), but it presented the drawback of an expandable, but still limited, field of view, due to its fixture in constraining the probe motion to pivoting about a single axis [20]. 

Our 3D ultrasound scanning method is based on a closed platform, a standard ultrasound machine. The 3D ultrasound reconstruction is generated from the DICOM files and the data from the coordinate measuring machine (CMM), an articulated arm (as the spatial pose reading sensor). The synchronization method and algorithms of the two data flows were developed in house. This constitutes another advantage compared to other developed methods, because direct data access is typically not enabled by commercial diagnostic systems and, thus, requires the development of open platforms or close collaborations with manufacturers for integration [11].

Other studies [21] measured the difference in the length from the surface of a phantom to the bottom part, using the ultrasound image, and found it to be 6.48 mm. At the same time, the difference in the position data from the ultrasonic sensor was 5.85 mm. The difference between the measured ultrasound image and the position data was only 0.65 mm (9.72%) [21]. 

A scanner with three translational degrees of freedom was used in another study to scan the teeth from an occlusal direction. One tooth per scan was 3D ultrasound reconstructed. The mean difference between the reconstructed casts and the optical control group was in the range 14–53 µm. The standard deviation was between 21 and 52 µm [22]. 

Comparing the aforementioned results with the ones obtained in the present study, a similar or better accuracy can be noted in the current study, regarding the mean difference. The overall mean distance ranged between 0.014 mm and 0.050 mm and the standard deviation ranged between 0.287 mm and 0.565 mm. The standard deviation was higher in our case because of the freehand scanning technique, which generated higher artifacts in the acquired 2D ultrasound images. The homogenous distribution of the errors at the scanned area level, the extension of the scanned area, six teeth instead of a single tooth scan and the Gaussian distribution of the errors generate promising premises for the future evaluation and use of a freehand 3D ultrasonographic scanning method in clinical settings.

A study performed by Marotti et al. on extracted teeth covered with porcine gingiva reported mean deviations for 3D ultrasound scans, ranging from between 12.34 to 46.38 µm [23]. 

The performance of intraoral optical scanners, reported by Winkler et al. in their study about TRIOS 3, displayed slightly higher precision (approximately 10 μm) compared to CS 3600, only after superimposition on the whole dental arch (*p* < 0.05). Both intraoral scanners showed good performance and comparable trueness (median of 0.0154 mm; *p* > 0.05) [24]. Comparing their results with our 3D ultrasound imaging method showed lower precision on our side, mostly due to the artefacts and noise in the acquired 2D ultrasound images. The precision of our pose reading sensor device was a maximum of 25 μm, according to the manufacturer’s technical specifications. This also contributed to the accumulated error, which ranged between 14 and 50 μm for the 3D ultrasound reconstructions, compared to the CAD reference object. In addition, the CAM of the reference scanned object has induced errors of at most 7 μm, according to the technical specifications of the device. 

The following are the main limitations of the current study: the 3D ultrasound reconstructions were performed only for the surfaces of the scanned object. Comparing the results with the STL CAD project of the object allowed the appreciation of the accuracy (trueness and reproducibility) of the scanning method. Future studies should also evaluate the prototype’s scanning accuracy of deep soft tissue and bone surfaces. Another limitation of the current study drew from the fact that only a single object had been scanned. In future studies, we intend to evaluate the proposed prototype by scanning different patients and different types of tissue, so that the segmentation process will also be challenged by the need to correctly identify the anatomical parts or pathological tissues.

## 5. Conclusions

The quantitative evaluation of the proposed 3D ultrasonographic reconstruction method showed comparable results to other studies performed on smaller areas of scanned objects, thus, demonstrating the future potential of the developed prototype to be used in clinical practice. The freedom of movement during scanning and the accuracy of the 3D reconstructions will have to be exploited in future research to evaluate and monitor the evolution of diseases, by comparing the 3D models. This process can be performed by integrating automatic or semi-automatic methods for the segmentation and alignment of the 3D objects.

## Figures and Tables

**Table 1:** Statistical analysis: mean distance and standard deviation for the 3D ultrasonographic point clouds of six consecutive scans of the same CAD/CAM manufactured object.

Scan	Range of 2D Ultrasound Frames	Segmentation Mode	Scanning Time	Number of 3D Points	Mean Distance	Std Deviation
1	300–752	Segmentation without contour extraction	13.69 s	279,189	0.033 mm	0.387 mm
2	232–611	Segmentation and contour extraction	11.48 s	46,537	0.031 mm	0.287 mm
3	252–703	Segmentation and contour extraction	13.66 s	65,535	0.050 mm	0.350 mm
4	260–779	Segmentation and contour extraction	15.72 s	70,774	0.014 mm	0.352 mm
5	220–674	Segmentation and contour extraction	13.75 s	54,378	0.023 mm	0.372 mm
6	400–979	Segmentation and contour extraction	17.54 s	76,735	0.019 mm	0.565 mm
